# Supplementary material for: Multivalley engineering in semiconductor microcavities
Source: Sci Rep. 2017 Apr 3;7:45243. doi: 10.1038/srep45243 (PMC5377251; doi:10.1038/srep45243)
Supplement: Supplementary Information [file srep45243-s1.pdf]

Supplementary Material for "Multivalley engineering in semiconductor microcavities"

M. Sun[1*], I. G. Savenko[1,2,3], H. Flayac[4], and T. C. H. Liew[5]

[1]Center for Theoretical Physics of Complex Systems, Institute for Basic Science, Daejeon, Republic of Korea
[2]Nonlinear Physics Centre, Research School of Physics and Engineering, The Australian National University, Canberra ACT 2601, Australia
[3]ITMO University, St. Petersburg 197101, Russia
[4]Institute of Theoretical Physics, Ecole Polytechnique Fédérale de Lausanne (EPFL), CH-1015 Lausanne, Switzerland
[5]Division of Physics and Applied Physics, School of Physical and Mathematical Sciences, Nanyang Technological University, 21 Nanyang Link, Singapore 637371
[*]sunmeg.89@gmail.com

ABSTRACT

In this Supplementary we present details of calculations for the main text of the Report.

Bloch theory for exciton-photon lattices

Here, we present the calculation of bare exciton-polariton (EP) dispersion in the framework of the Bloch theory. In the main text of the manuscript, we solve the eigenvalue problem of the system, Eq. (1). This equation corresponds to the eigenvalue problem of the following matrix:

$$\begin{pmatrix} \tilde{E}_C(k+G)+\tilde{V}_C(0) & \tilde{V}_C(G) & \tilde{V}_C(2G) & 0 & 0 & \Omega \\ \tilde{V}_C(-G) & \tilde{E}_C(k)+\tilde{V}_C(0) & \tilde{V}_C(G) & 0 & \Omega & 0 \\ \tilde{V}_C(-2G) & \tilde{V}_C(-G) & \tilde{E}_C(k-G)+\tilde{V}_C(0) & \Omega & 0 & 0 \\ 0 & 0 & \Omega & \tilde{E}_X(k-G)+\tilde{V}_X(0) & \tilde{V}_X(G) & \tilde{V}_X(2G) \\ 0 & \Omega & 0 & \tilde{V}_X(-G) & \tilde{E}_X(k)+\tilde{V}_X(0) & \tilde{V}_X(G) \\ \Omega & 0 & 0 & \tilde{V}_X(-2G) & \tilde{V}_X(-G) & \tilde{E}_X(k+G)+\tilde{V}_X(0) \end{pmatrix}, \tag{1}$$

where $\tilde{E}_C(q)=\frac{\hbar^2 q^2}{2m_C}-\frac{i\hbar}{\tau}$, $\tilde{E}_X(q)=\frac{\hbar^2 q^2}{2m_X}-\frac{i\hbar}{\lambda}$, $\tilde{V}_C(0,-G,G)$ are the 0th, -1st, 1st order terms of the Fourier series for the periodic potential of the cavity photon. $\tilde{V}_X(0,-G,G)$ are the 0th, -1st, 1st order terms of the Fourier series for the excitonic potential. The matrix in (1) is reduced to the summation of terms in Eq. (1) from $-G$ to G. In our real calculations, we did the summation over the terms from $-150\cdot G$ to $150\cdot G$.

Simplified model of equilibrium polariton condensation

Following Eqs. (2) and (3) in the main text, for the case of a fixed number of particles and small temperature, we can write the probability of occupation of different modes referred to as the probability distribution function (PDF):

$$p(n_1,n_2)\,\frac{1}{\mathscr{Z}}e^{-E(n_1,n_2)/k_BT}, \tag{2}$$

where $\mathscr{Z}=\sum_{n_1,n_2}e^{-E(n_1,n_2)/k_BT}$ is the partition function, T is the temperature and k_B is Boltzmann's constant. The second order correlation function then reads

$$g^{(2)}_{12}=\frac{\langle\hat{a}_1^\dagger\hat{a}_2^\dagger\hat{a}_1\hat{a}_2\rangle}{\langle\hat{a}_1^\dagger\hat{a}_1\rangle\langle\hat{a}_2^\dagger\hat{a}_2\rangle}=\frac{\langle n_1n_2\rangle}{\langle n_1\rangle\langle n_2\rangle}, \tag{3}$$

where $\langle n_1 \rangle$, $\langle n_2 \rangle$, and $\langle n_1 n_2 \rangle$ can be calculated from the PDF, $p(n_1, n_2)$, see Fig. 2 in the main text. At zero temperature $g_{12}^{(2)} = 0$, confirming our earlier arguments on the choice of the state required for energy minimisation. With increasing temperature, $g_{12}^{(2)}$ rises as the system may be excited out of the ground state.

Allowing for the population of many modes in reciprocal space (instead of the only two which we considered), the probability of occupation of any quantum state can be found by straightforward generalisation of Eq. (2). In principle, the full EP intensity distribution can then be obtained by summing over the PDF. However, in practice, the size of the Hilbert space grows exponentially with the number of particles in the system, therefore, it becomes possible to use our simple treatment to evaluate the equilibrium photoluminescence spectrum in the low density regime only with a few particles in the system (see also inset in Fig. 2 in the main text). The spectrum here was phenomenologically broadened in energy and wave vector, accounting for the finite lifetime of polaritons and finite size of a typical condensate, respectively.

Entanglement generation

We treat particles localized at the dispersion minima as two quantum modes, which deterministic evolution is governed by the Hamiltonian (5) in the main text. The last term there, $\hat{\mathscr{H}}'_{CK} = 4\alpha \hat{a}_1^\dagger \hat{a}_2^\dagger \hat{a}_2 \hat{a}_1$, is a typical cross-Kerr interaction term that can be linearised by expanding the total quantum fields as $\hat{a}_j \to \xi_j + \delta\hat{a}_j$, where $\delta\hat{a}_j$ are the displaced quantum fluctuation fields on top of the classical mean fields, ξ_j. Using such a substitution and keeping terms up to the second order in $\delta\hat{a}_j$ only, we come up with the linearised interaction term,

$$\hat{\mathscr{H}}'^{(\mathrm{lin})}_{CK} = 4\alpha \sum_{j=1,2} \left[\tilde{E}_j \delta\hat{a}_j^\dagger \delta\hat{a}_j + \tilde{F}_j (\delta\hat{a}_j^\dagger + \delta\hat{a}_j) \right] + \alpha\mathscr{S} \left(\delta\hat{a}_1^\dagger \delta\hat{a}_2^\dagger + \delta\hat{a}_1 \delta\hat{a}_2 \right), \tag{4}$$

where we assumed $\xi_{1,2} \in \mathbb{R}$ for clarity and defined $\tilde{E}_{1,2} = \Delta_{2,1}|\xi_{1,2}|^2$, $\tilde{F}_{1,2} = \xi_{1,2}|\xi_{2,1}|^2$ and $\mathscr{S} = \xi_1\xi_2$. The first term is an energy shift, the second one is an effective driving term, and crucially, the last term is a typical two-mode squeezing interaction of magnitude $\mathscr{S}$ set by the product of the two mean fields, $\xi_{1,2}$, and responsible for the entanglement between the two modes. The squeezing and entanglement magnitude can therefore be adjusted by varying the resonant driving strengths, $F_{1,2}$.

Nonequilibrium model of polariton condensation

The interaction with the reservoir of acoustic phonons of the semiconductor crystal lattice is described by the microscopic Fröhlich Hamiltonian:[1]

$$\mathscr{H}_{\mathrm{int}} = \sum_{\mathbf{q},k} G_{\mathbf{q}} \hat{b}_{\mathbf{q}} \hat{a}^\dagger_{k+q_x} \hat{a}_k + G^*_{\mathbf{q}} \hat{b}^\dagger_{\mathbf{q}} \hat{a}_{k+q_x} \hat{a}^\dagger_k, \tag{5}$$

where parameters $G_{\mathbf{q}}$ are the exciton-phonon interaction strengths evaluated elsewhere[2]. The phonon wavevector here is $\mathbf{q} = \mathbf{e}_x q_x + \mathbf{e}_y q_x + \mathbf{e}_z q_z$, where $\mathbf{e}_x$, $\mathbf{e}_y$ and $\mathbf{e}_z$ are unit vectors: $\mathbf{e}_x$ is in the direction of the 1D polariton system, $\mathbf{e}_z$ is in the structure growth direction, and $\mathbf{e}_y$ is perpendicular to both of those two. The phonon dispersion relation, $\hbar\omega_{\mathbf{q}} = \hbar c_s \sqrt{q_x^2 + q_y^2 + q_z^2}$, is determined by the sound velocity, c_s.

The equations of motion for the polariton macroscopic wave function, ψ, and the exciton reservoir occupation number, n_{R}, read[3,4]:

$$\begin{aligned} i\hbar \frac{\partial \psi(x,t)}{\partial t} &= \mathscr{F}^{-1}\left[E_k \psi_k + \mathscr{S}_k(t)\right] + \frac{i\hbar}{2}\left[R n_{\mathrm{R}}(x,t) - \gamma_0 - \frac{2i}{\hbar}\alpha|\psi(x,t)|^2\right]\psi(x,t) \\ &\quad + \sum_k \left[\mathscr{T}_{-k}(t) + \mathscr{T}^*_k(t)\right] e^{-ikx}\psi(x,t); \end{aligned} \tag{6}$$

$$\frac{\partial n_{\mathrm{R}}(x,t)}{\partial t} = -(\gamma_{\mathrm{R}} + R|\psi(x,t)|^2) n_{\mathrm{R}} + P, \tag{7}$$

where $\mathscr{F}^{-1}$ stands for the inverse Fourier transform, E_k is the free polariton dispersion, ψ_k is the Fourier image of the macroscopic wave function; P and γ_{R} are the incoherent reservoir homogenous pumping intensity and inverse lifetime of the reservoir, correspondingly; R is the system-reservoir excitation exchange rate. The term $\mathscr{S}_k(t)$ corresponds to the emission of phonons by a condensate stimulated by the polariton concentration.

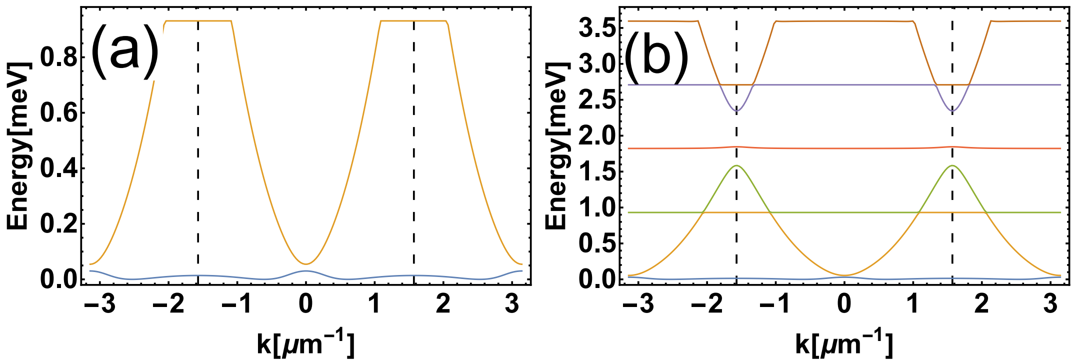

Figure 1. Energy bands corresponding to Fig. 1 in the main text: (a) level 1 and level 2. (b) from level 1 to level 6.

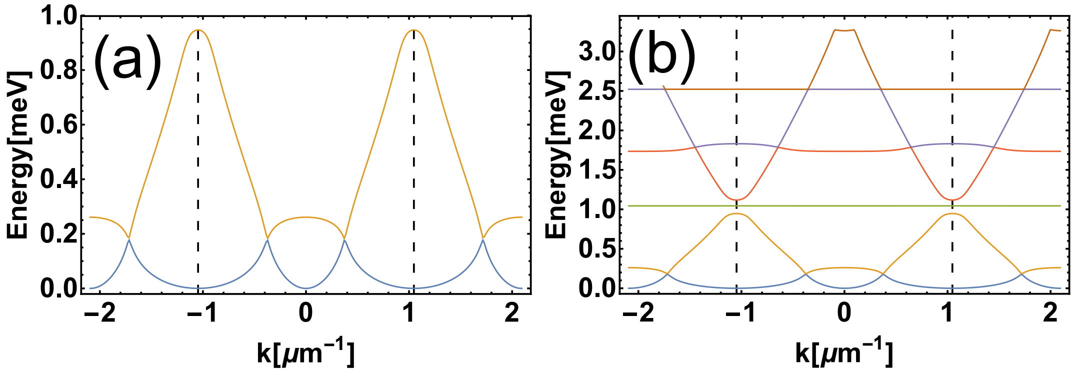

Figure 2. Energy bands in the case when the minima in k-space are at the Γ point and the edge of the first BZ: (a) level 1 and level 2. (b) from level 1 to level 6.

The stochastic term $\mathscr{T}_{q_x}$ in the last line of (6) is defined by the correlations:

$$\left\langle \mathscr{T}^*_{q_x}(t)\mathscr{T}_{q'_x}(t')\right\rangle = \sum_{q_y,q_y} \left|G_{q_x,q_y,q_z}\right|^2 n_{q_x,q_y,q_z}\delta_{q_x,q'_x}\delta(t-t');$$
$$\left\langle \mathscr{T}_{q_x}(t)\mathscr{T}_{q'_x}(t')\right\rangle = \left\langle \mathscr{T}^*_{q_x}(t)\mathscr{T}^*_{q'_x}(t')\right\rangle = 0, \tag{8}$$

where $n_{\mathbf{q}}$ is the temperature-dependent density of phonons in the state with a wave vector $\mathbf{q}$.

Solving (6) numerically, and averaging over different stochastic realizations of the phonon field, we obtain the results shown in Fig. 3 of the main text.

Energy band structure

Here we present details on the energy band structure calculation for the EP lattice, see Fig. 1a in the main text and one more alternative configuration. Parameters of these plots are: the lattice period: $T = 2.0$ μm, potential profile for the cavity photons: the sine function $-1.1 \sim 0.25$ meV, potential profile for the excitons: the sine function $-0.95 \sim 460.29$ meV, exciton-photon coupling constant: $\Omega = 0.7$ meV, decay rate for the cavity photons: $\gamma = 0.42$ meV, decay rate for the excitons: 0.04meV, effective mass of the cavity photon: $5*10^{-5}m_e$, effective mass of the exciton: $0.22m_e$, where m_e is the free electron mass.

Let us also change the parameters of the potential energies of excitons and photons and see, which dispersion we can achieve in k-space. If we take the lattice period: $T = 3.0$ μ m, potential profile for cavity photon: square function $-0.25 \sim 0$ meV, potential profile for exciton: sine function $-0.389 \sim 697.95$ meV, exciton-photon coupling constant: $\Omega = 2.47$ meV, decay rate for cavity photon: $\gamma = 1$ meV, decay rate for exciton: 0 meV, effective mass of cavity photon: $5*10^{-5}m_e$, effective mass of exciton: $0.22m_e$, where m_e is the free electron mass, then we yield the dispersion presented in Figs. 2 and 3.

Figure 3 is the result of the nonequilibrium model. Since the decay rate of the EPs vary significantly with momentum k, it becomes easier for EPs to condensate at $k = \pm k_{BZ}$ rather than $k = 0$. As a result, the blueshifts are different at $k = 0$ and at

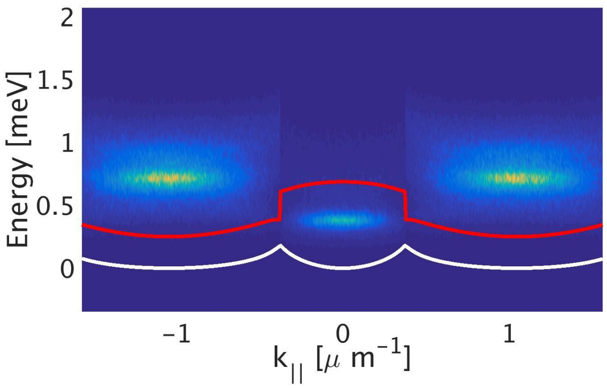

Figure 3. Formation of the BEC (condensation) calculated for the energy dispersion presented in Fig. 2. The red line shows the profile of the decay rate of EPs. The white line is the energy dispersion.

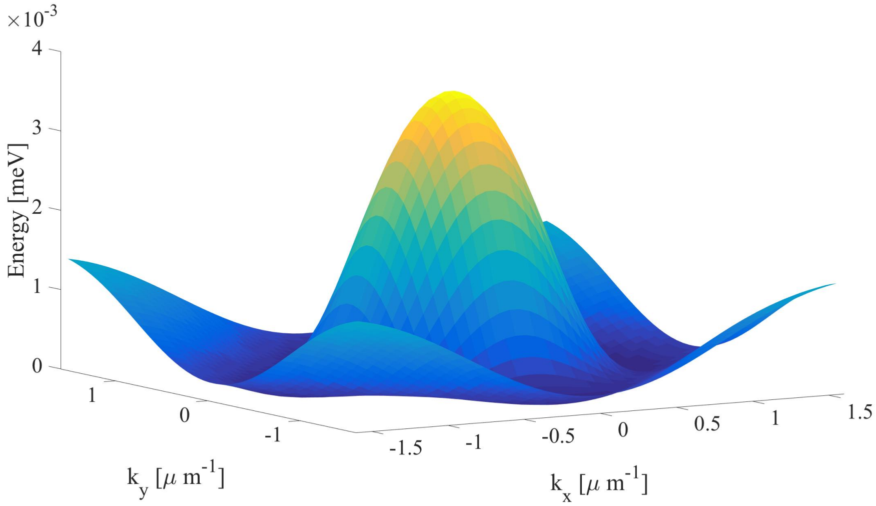

Figure 4. The energy dispersion for the two-dimensional lattice.

$k = \pm k_{BZ}$. The minima in the k-space become inequivalent in properties, making it difficult to consider entanglement between them. It should be noted, that, instead, in Fig. 3b in the main text the two minima at $k = \pm k_0$ are equivalent in properties.

Figure 4 is the 3D plot of the energy dispersion in 2D lattice corresponding to Fig. 5a in the main text. One can see four degenerate valleys with equivalent properties. It results in the possibility to create spin-valley coupling.

References

1. Tassone, F., Piermarocchi, C., Savona, V., Quattropani, A. & Schwendimann, P. *Phys. Rev. B*, **56**, 7554 (1997).

2. Hartwell, V. E., & Snoke, D. W. *Phys. Rev. B*, **82**, 075307 (2010).

3. Wouters, M. & Carusotto, I. *Phys. Rev. Lett.* **99**, 140402 (2007).

4. Savenko, I. G., Liew, T. C. H. & Shelykh, I. A. *Phys. Rev. Lett.* **110**, 127402 (2013).
